# Evaluation of the Effectiveness of Hyodine on Healing of Diabetic Foot Ulcer: A Randomized Single-Blind Controlled Trial

**DOI:** 10.2174/0118715303426166251203081819

**Published:** 2026-05-07

**Authors:** Rezvan Razmandeh, Faeze Abbaspour, Leila Yazdanpanah, Samimeh Shahbazi, Mohammadreza Heidari, Ozra Tabatabaei-Malazy, Mohammadreza Amini

**Affiliations:** 1 Diabetes Research Center, Endocrinology and Metabolism Clinical Sciences Institute, Tehran University of Medical Sciences, Tehran, Iran;; 2 Non-Communicable Diseases Research Center, Endocrinology and Metabolism Population Sciences Institute, Tehran University of Medical Sciences, Tehran, Iran;; 3 Health Research Institute, Diabetes Research Center, Ahvaz Jundishapur University of Medical Sciences, Ahvaz, Iran;; 4 Endocrinology and Metabolism Research Center, Endocrinology and Metabolism Clinical Sciences Institute, Tehran University of Medical Sciences, Tehran, Iran

**Keywords:** Diabetic foot ulcer, controlled trial, Hyodine, type 1 and 2 diabetes, antimicrobial activity, hyaluronic acid

## Abstract

**Introduction:**

Diabetic foot ulcers (DFUs) pose a significant global health threat. It is estimated that individuals with diabetes have a 15–34% lifetime risk of developing a foot ulcer. This clinical study evaluates the comparative effectiveness of Hyodine solution *versus* standard treatment protocols in promoting the healing of DFUs.

**Methods:**

This prospective, multicenter, single-blind randomized clinical trial enrolled 98 patients with type 1 and 2 diabetes who presented with grade 1-2 infected diabetic foot ulcers from participating diabetes clinics between June 2023 and January 2025. Patients were randomly divided into two groups by block randomization in a 4:1 ratio: a control group that received standard treatment and an intervention group (Hyodine) that received standard treatment plus an iodine solution. Wound cultures were obtained three times during the study: at baseline (before treatment initiation), week 2, and week 4. The collected data were analyzed using SPSS 20 software.

**Results:**

In our study, 98 patients with diabetic foot ulcers participated. Intervention group (n = 49): received Hyodine treatment, and the control group (n = 49) received standard wound care, for four weeks. The average age of the participants was 60 ± 11.46 years. Among the participants, 30 patients (30.6%) were females, and 63.3% of the individuals had a history of diabetic foot ulcer. The colony counts significantly decreased by the end of the study compared to the beginning of the study (*P*<0.001) in the intervention group. The difference in wound area in the Hyodine group between the first and last visits was statistically significant (*P* < 0.05).

**Discussion:**

These results align with growing evidence that hyaluronic acid-based treatments, such as Hyodine, enhance antimicrobial activity and accelerate wound healing in chronic diabetic foot ulcers.

**Conclusion:**

To reduce complications from diabetic foot ulcers, the implementation of advanced therapeutic interventions is critical for alleviating the burden on both patients and healthcare systems.

**Clinical Trial Number:**

IRCT20088094001199N8.

## 1 INTRODUCTION

### 1.1 Global Relevance and Epidemiology

Diabetic foot ulcers (DFUs) represent a significant and growing global health challenge. According to the Global Burden of Diseases Study (GBD), the number of people living with diabetes worldwide is projected to rise to 1.31 billion by 2050 [1], with global healthcare expenditures related to diabetes forecast to surge to USD 1.054 trillion by 2045 [2]. It is estimated that individuals with diabetes face a 15–34% lifetime risk of developing a foot ulcer. In Iran, specifically, the National Program for the Prevention and Control of Diabetes estimates the prevalence of DFU at 6.2% [3, 4]. These lower limb complications are a leading cause of hospitalization, resulting in substantial financial and personnel costs for healthcare systems worldwide [5, 6].

### 1.2 Pathophysiology and Complications

The pathogenesis underlying the development and persistence of Diabetic Foot Ulcers (DFUs) is multifactorial. A significant challenge is the coexistence of peripheral neuropathy, which leads to a loss of protective sensation, and ischemia, which impairs blood flow and healing capacity. This environment is frequently complicated by infection, with diabetes-related foot infections affecting approximately 40% of foot ulcers. These infections are a primary contributor to patient morbidity, greatly increasing the risk of hospitalization, lower extremity amputation, and mortality [7].

Diabetic foot osteomyelitis is a serious bone infection that commonly arises as a complication of diabetic foot ulcers or soft tissue infections. It often develops when bacteria spread from a chronic ulcer or wound to the underlying bone, especially in diabetic patients with neuropathy and poor circulation, which impair wound healing. If left untreated, it significantly increases the risk of limb amputation and prolongs hospital stays and healthcare costs. Early diagnosis and management are critical to reduce morbidity and improve patient outcomes.

### 1.3 Modern Treatments and Emphasis on Hydrogen

Modern DFU management focuses on addressing these multifaceted challenges through advanced wound care. One such approach involves dressings containing hyaluronic acid (HA), which stimulate angiogenesis, maintain a moist wound environment, reduce exudate, and promote tissue repair [8]. Sodium hyaluronate, a key glycosaminoglycan, is central to these processes due to its role in inflammation, cell proliferation, and wound healing [9, 10]. Hyodine is a novel solution that combines sodium hyaluronate with potassium iodide and iodine. This combination is designed to leverage the regenerative properties of hyaluronate while mitigating the risks associated with iodine alone, which can be cytotoxic to healthy tissue and delay healing. The iodine component enhances broad-spectrum antiseptic efficacy and protects the sodium hyaluronate from bacterial denaturation [11, 12].

The beneficial effects of its components have been demonstrated in other wound models, including high success rates in the management of gastric bleeding [13-15]. Therefore, this randomized controlled trial aims to investigate the efficacy of Hyodine solution specifically in the treatment of diabetic foot ulcers.

## 2 MATERIALS AND METHODS

### 2.1 Study Design and Participants

A prospective, multicenter, single-blind randomized clinical trial was conducted from June 2023 to January 2025. The study enrolled 98 diabetic patients (type 1 and type 2) with grade 1 or 2 infected diabetic foot ulcers from two centers in Iran: The Endocrine and Metabolism Research Institute of Tehran University of Medical Sciences and the Diabetic Foot Clinic of Ahvaz. A uniform protocol was followed at both centers.

### 2.2 Inclusion and Exclusion Criteria

Eligible participants were individuals aged 30 to 75 years with a DFU classified as neuropathic or neuro-ischemic, categorized as grade 1 or 2 according to both the PEDIS (Perfusion, Extent, Depth, Infection, Sensation) [16] and Wagner [17] classification systems. Participants were required to have a hemoglobin A1C (HbA1c) level below 9%. Exclusion criteria included unwillingness to participate, a history of skin allergies, coagulopathy, or thyroid diseases. All patients provided written informed consent.

### 2.3 Randomization and Blinding

Participants were randomized using block randomization (block size of four) *via* an online tool (SealedEnvelope) to ensure unbiased allocation. This generated a random numeric code sequence, with participants assigned codes in the order of their enrollment. The study employed a single-blind design. The treating physicians, nurses responsible for measuring wound size and obtaining cultures, microbiology laboratory staff, and the statistical analyst were all blinded to group assignments. Blinding of the nurses applying the dressings and the participants was not possible due to the visible differences between the interventions (Fig. **1**) [18].

### 2.4 Interventions

Following randomization, 49 patients were assigned to the control group and 49 to the intervention group. All patients received sharp debridement and standard offloading care.

Control Group (SilvoSept Wound Spray [19]): This group received standard wound care, which consisted of a topical antiseptic containing nano-colloidal silver particles (SilvoSept, Chitotech Company). The solution's mechanism of action is based on the oligodynamic effect of silver ions, which provide broad-spectrum antimicrobial activity against a wide range of bacteria. It was applied *via* spray as three puffs directly onto the wound bed and peri-wound skin during each dressing change.

Intervention Group: This group received standard wound care plus the application of the investigational product (Hyodine).

This is a topical gel foam composed of sodium hyaluronate (0.5% w/w), potassium iodide, and iodine (0.1% w/w). The formulation is designed to combine the wound-healing properties of hyaluronic acid—which promotes angiogenesis, facilitates cell migration, and maintains a moist wound environment—with the broad-spectrum antiseptic action of iodine. The iodine component is stabilized within the hyaluronic acid matrix to mitigate potential cytotoxicity to regenerating tissue. It was applied topically to the wound bed at a volume of approximately 1 ml per 10 cm^2^ of wound area during each session.

Dressing changes for both groups were performed three times per week at 48-hour intervals.

### 2.5 Data Collection

Wound cultures were obtained at three time points: baseline (before treatment initiation), week 2, and week 4. Samples were collected by pressing and rotating a cotton swab within a one-square-centimeter area of the wound's depth, placed in transport medium, and cultured on blood agar and Tryptone Soya Agar (TSA) for qualitative and semi-quantitative analysis (MIRMEDIA [20]. Wound size was measured using a ruler and verified with ImageJ software. Adverse effects were monitored and recorded using a case report form (CRF) questionnaire throughout the study period and at the final week 4 visit.

Hyodine^®^ is a medical gel foam composed of hyaluronic acid and iodine (Nikta: Nikan Teb Kimia Company). It should be stored at temperatures up to 25°C. Once opened, the product must be kept in a cool place (2-8°C), protected from direct sunlight and freezing. Before application, Hyodine^®^ should be brought to room temperature. The method of using Hyodine^®^ was explained to nurses and patients as follows:

- Pierce the vial cap with the attached spike and connect the sterile syringe.
- Draw the required amount of gel into the syringe (approximately 1 ml per 10 cm^2^ of wound).
- Apply directly to the wound bed or to a carrier dressing (non-woven fabric or sterile gauze). Do not use on black necrotic tissue.
- Work Hyodine^®^ into the carrier with your fingers until it becomes well saturated.
- Cover with a suitable secondary dressing.
- Depending on the condition of the wound, re-dress every 24 hours for infected wounds or every 48 hours for non-infected wounds.

### 2.6 Ethical Considerations

The study protocol was approved by the Research Ethics Committee of the Endocrine & Metabolism Research Institute - Tehran University of Medical Sciences (Approval ID: IR.TUMS.EMRI.REC.1402.038) and registered in the Iranian Registry of Clinical Trials (code: IRCT20088094001199N8).

### 2.7 Statistical Analysis

Descriptive analyses are presented as means ± standard deviations for continuous variables and as frequencies and percentages for categorical variables. Comparisons of continuous variables between groups were performed using T-tests and Repeated Measures ANOVA. All analyses were conducted using SPSS software version 22, with a statistical significance level set at *P* < 0.05.

## 3 RESULTS

Our study included 98 patients with diabetic foot ulcers, divided equally into two groups: 49 patients (50%) received Hyodine treatment, and 49 patients (50%) received standard care (Silver Spray) as the control group. Among the participants, 30 (30.6%) were female and 68 (69.4%) were male, with 86% being married. The average age of the participants was 60 years. Regarding occupational status, 30.6% were employed and 6.6% were unemployed. Most participants (55.1%) had a high school diploma or lower education level. Half of the patients had a history of hospitalization due to diabetes-related complications, and 63% had a prior history of foot ulcers. Lower limb examinations revealed abnormalities in 69.4% of participants, including skin conditions, nail deformities (26.5%), and foot deformities (40.8%). All participants had type 2 diabetes, with an average disease duration of 18.5 years (Table **1**).

Laboratory parameters were recorded, including fasting blood glucose (168.8 ± 70.4 mg/dL) and HbA1c levels (8.3% ± 1.487). The severity of the wounds was classified using international grading systems: Wagner, PEDIS, and SINBAD (Table **2**).

We evaluated microbial colony counts in the wounds. As shown in Table **3** and Fig. (**2**), there was a significant decrease in infection rates from the first to the last wound cultures. The difference in mean bacterial colony counts between the Hyodine and control groups was statistically significant (*P* < 0.001).

The results revealed that the average initial wound size was 3.2 cm^2^ in the Hyodine group and 2.6 cm^2^ in the control group. At the final assessment, the Hyodine group showed a reduced wound size, decreasing to 1.2 cm^2^, while the standard treatment group had a smaller reduction to 2.2 cm^2^ (Fig. **3**). The difference in wound size between the first and final assessments in the Hyodine group was statistically significant (*P* < 0.05).

## 4 DISCUSSION

Our findings indicated that both wound area and bacterial colony count significantly decreased after 4 weeks of intervention with Hyodine plus standard care, compared to standard care alone.

Vaezi *et al*. studied 620 diabetic patients and demonstrated that regular follow-up for diabetic foot ulcers significantly improved wound healing and prevented new ulcers. This finding aligns with the present study, which shows that regular patient follow-ups, weekly debridement, patient education, offloading, and proper footwear lead to clinically meaningful improvements in outcomes when combined with new therapies. These benefits were observed in both the Hyodine and standard treatment groups [21].

A prospective study of 71 patients with severely infected chronic diabetic foot ulcers demonstrated the efficacy of a new sodium hyaluronate-impregnated dressing. Their findings indicated that optimal wound healing requires three key factors: (1) effective infection and inflammation control, (2) consistent follow-up, and (3) application of this specialized dressing. The sodium hyaluronate layer has antimicrobial properties that combat common pathogens such as *Staphylococcus aureus* and *Pseudomonas aeruginosa*. It also decreases wound exudate and creates an optimal microenvironment for granulation tissue formation. This dual mechanism facilitates the removal of inflamed tissue while promoting angiogenesis and tissue regeneration [9, 22]. Our research supports these results, demonstrating both microscopic and macroscopic wound healing over a five-week period, which included weekly Hyodine use, regular debridement, and detailed photographic documentation at every follow-up.

A recent meta-analysis established hyaluronic acid as a critical modulator across all phases of wound healing. In the inflammatory phase, it binds to fibrinogen to initiate the clotting pathway, facilitates the migration of inflammatory cells, induces edema to support cellular infiltration, and inhibits neutrophil migration to reduce excessive inflammation [23]. In the granulation phase, hyaluronic acid promotes cell mitosis, enhances cellular migration, and stimulates angiogenesis. During epithelial reconstruction, it supports keratinocyte proliferation and migration. In the remodeling phase, it aids both normal and pathological wound healing. Additionally, hyaluronic acid acts as a scavenger of free radicals and tissue-degrading enzymes that contribute to prolonged inflammation in chronic wounds [24, 25]. Beyond moisture regulation, our clinical observations indicate that Hyodine promotes faster granulation, epithelialization, and infection control in chronic diabetic foot ulcers, leading to significant wound size reduction.

Other studies have reported that hyaluronic acid decreases exudate production and reduces maceration around wounds. By creating a favorable, infection-free environment, it enhances the migration of healing cells and supports proper angiogenesis, thereby aiding the healing of chronic wounds [25-27]. In our weekly follow-ups, we observed a progressive reduction and eventual disappearance of exudate and inflammation, along with improvements in wound edges and the wound bed, ultimately resulting in the healing of diabetic foot ulcers.

A recent study involving 56 patients with diabetic foot ulcers ranging from mild to moderate infection showed that hyaluronic acid had comparable effectiveness to hypochlorous acid disinfectant spray against Gram-positive bacteria (*P* < 0.05). Significant differences were also observed in average wound area and the inflammatory marker CRP between the two groups (*P* < 0.05) [28, 29]. In our investigation, most wounds were classified as Wagner grade II to III, and we observed a significant reduction in microbial colony counts followed by accelerated and improved wound healing by the final visit (week five) compared to the standard treatment group.

## 5 LIMITATIONS

The small number of participants may limit the generalizability of the findings to the broader population of patients with diabetic foot ulcers (DFUs).

## CONCLUSION

The integration of adjuvant therapies with standard DFU treatment demonstrates superior clinical efficacy compared to standard care alone. This study found that adding Hyodine to standard wound care significantly reduced bacterial burden and promoted wound healing, as shown by a statistically significant decrease in colony counts and a reduction in wound area. This combined approach enhances wound healing rates while reducing the risk of infection, underscoring its potential to improve patient outcomes and lessen the burden on healthcare systems.

## Figures and Tables

**Fig. (1):**
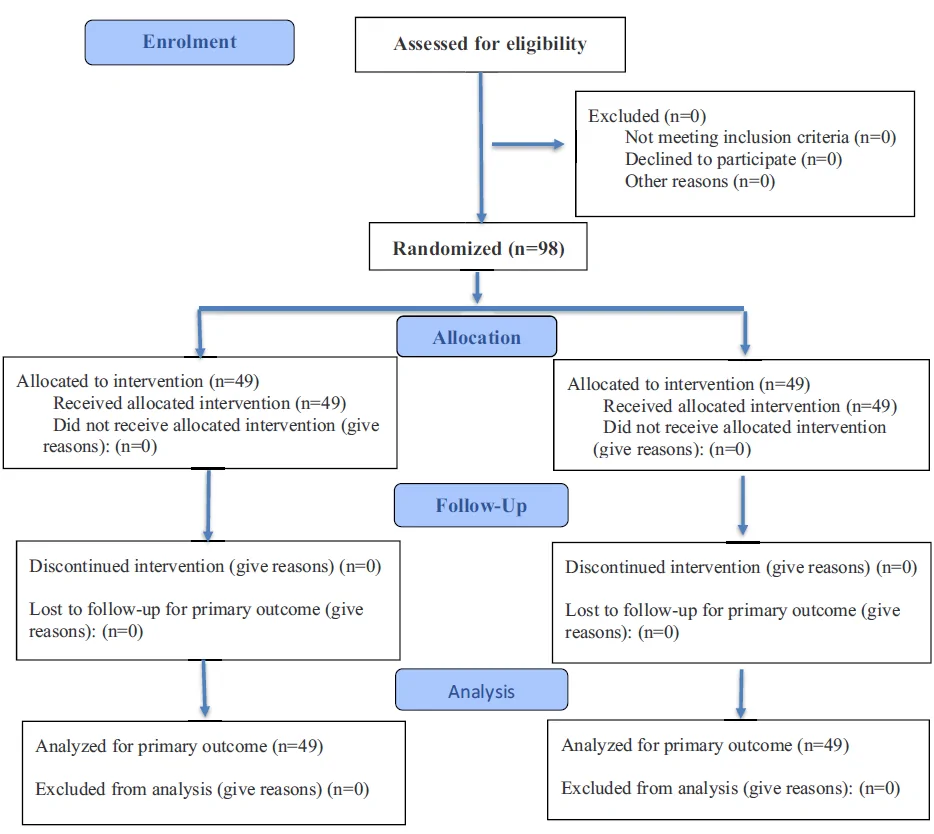
CONSORT 2025 flow diagram.

**Fig. (2):**
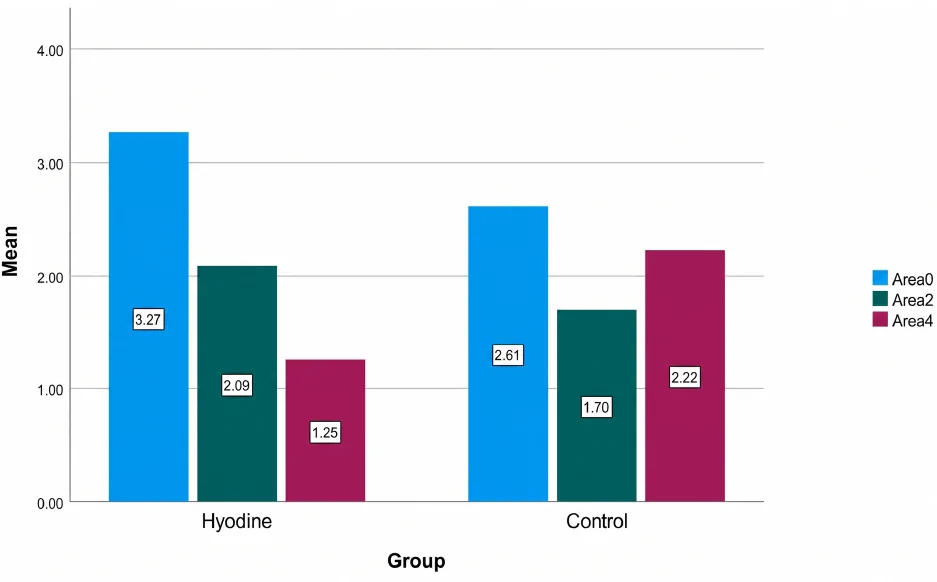
Mean wound area.

**Fig. (3):**
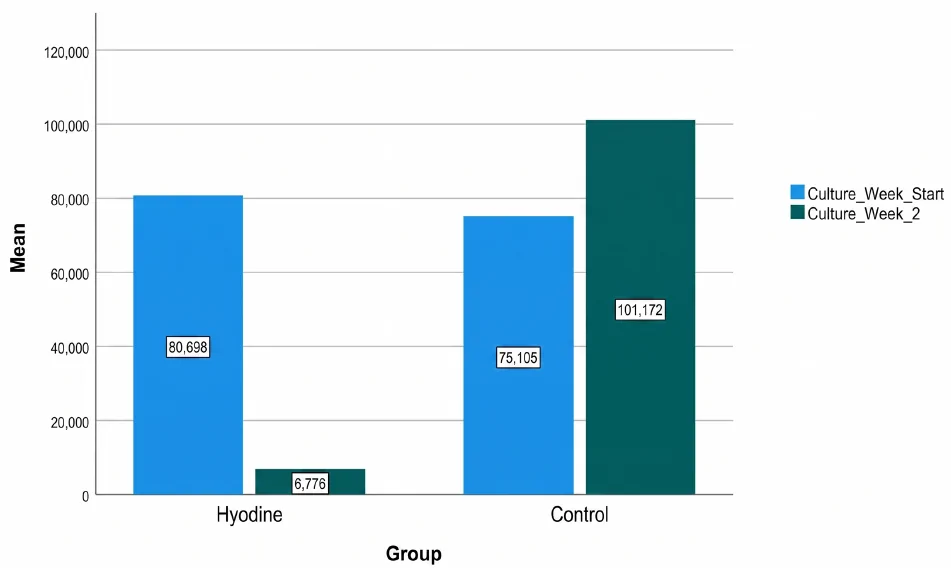
Mean of colony count.

**Table 1:** Demographic characteristics of the participants.

Hyodine	Age	49	35	80	60.31	11.468
	Duration of Diabetes	49	1	40	16.02	10.181
	FBS	49	80	388	158.14	60.523
	HbA1c	49	5.60	11.90	8.2837	1.55876
Control	Age	49	43	80	60.12	10.264
	Duration of Diabetes	49	2	75	21.10	11.762
	FBS	49	111	367	179.10	78.458
	HbA1c	49	5.20	11.00	8.3265	1.42840

**Table 2:** The severity of the wounds using international grading systems.

-		Frequency	Percent	Valid Percent	Cumulative Percent
Wagner	Superficial ulcer with exposed subcutaneous tissue	48	49.0	49.0	49.0
	Exposed tendons and deep structures	42	42.9	42.9	91.8
	Ulcers extend to the deep tissue and have either an associated soft tissue abscess or osteomyelitis	8	8.2	8.2	100.0
PEDIS	Grade2	60	61.2	61.2	61.2
	Grade3	38	38.8	38.8	100.0
SINBAD score	Low Severity	22	22.4	22.4	22.4
	Moderate Severity	48	49	49	71.4
	High Severity	28	28.6	28.6	100

**Legend:** PEDIS, SINBAD, Wagner.

**Table 3:** Decreasing the colony counts between the start week and the last week.

-	N	Minimum	Maximum	Mean	Std. Deviation
Culture _Week _Start	98	828	100000	77901.31	36555.819
Culture_Week_Last	98	0	698520	53974.15	121188.558

## Data Availability

All data generated or analyzed during this study are included in this published article.

## LIST OF ABBREVIATIONS

CRF: Case Report Form
DFUs: Diabetic Foot Ulcers
GBD: Global Burden of Diseases Study
HA: Hyaluronic Acid
HbA1c: Hemoglobin A1C
IRCT: Iranian Registry of Clinical Trials
PEDIS: Perfusion, Extent, Depth, Infection, and Sensation
SINBAD: Site, Ischemia, Neuropathy, Bacterial Infection, Area, and Depth
TSA: Tryptone Soya Agar

## STANDARDS OF REPORTING

CONSORT guidelines were followed.
